# Leiomyosarcoma of Inferior Vena Cava: Case Report

**DOI:** 10.1155/crra/9997163

**Published:** 2025-06-24

**Authors:** Johnny Lo, Khimseng Tew

**Affiliations:** ^1^Adelaide Medical School, University of Adelaide, Adelaide, South Australia, Australia; ^2^Department of Surgery, Royal Adelaide Hospital, Adelaide, South Australia, Australia; ^3^Department of Radiology, The Queen Elizabeth Hospital, Adelaide, South Australia, Australia

**Keywords:** cancer, case report, inferior vena cava, leiomyosarcoma, medical imaging, radiology, vascular

## Abstract

Leiomyosarcoma of inferior vena cava is a rare sarcoma subtype of vascular origin. It has a spectrum of clinical manifestations depending on the affected segment of inferior vena cava, which also determines the treatment and prognosis. We present a patient with metastatic leiomyosarcoma of inferior vena cava. Clinical presentation, radiological findings and management are discussed. In particular, we highlight the key features to consider on imaging to assist with preoperative planning.

## 1. Introduction

Vascular leiomyosarcomas are a rare sarcoma subtype and account for less than one in every 100,000 malignancies [1]. The most commonly involved blood vessel is inferior vena cava. There have only been less than 500 cases of leiomyosarcoma of inferior vena cava (LIVC) reported [1–3]. Following the first report of LIVC by Perl et al. in 1871 [2, 4], it was fifty years later until the first documented surgery occurred in 1928 [1].

Identifying the segment of IVC involvement is essential because it influences the clinical presentation, treatment and prognosis [1, 5]. We present a case of metastatic LIVC and review the current literature. We aim to highlight the key imaging features to assist radiologists when assessing a potential LIVC.

## 2. Case Report

A 47-year-old woman presented to the emergency department with a 2-week history of abdominal pain, recurrent fevers and bilateral leg swelling. Her medical history consisted of obesity and a previous hysterectomy for menorrhagia. On examination, she had lower abdominal tenderness, prompting a CT abdomen.

**Figure 1:**
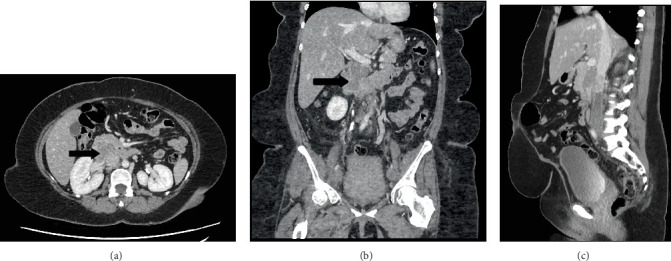
CT abdomen. (a) Axial, (b) coronal and (c) sagittal scans in portal venous phase show a heterogeneous mass (black arrow) within the region of the pancreatic head.

Abdominal CT with contrast (portal venous phase) showed a 61 × 40 × 28 mm heterogeneous mass in the region of pancreatic head with anterior displacement of adjacent duodenum (Figure 1). Extensive deep vein thrombosis was seen within the IVC, extending inferiorly to involve bilateral common, external and internal iliac veins and the visualised portion of femoral veins. A completion CT pulmonary angiogram was performed, which demonstrated multiple segmental pulmonary emboli but no metastases.

**Figure 2:**
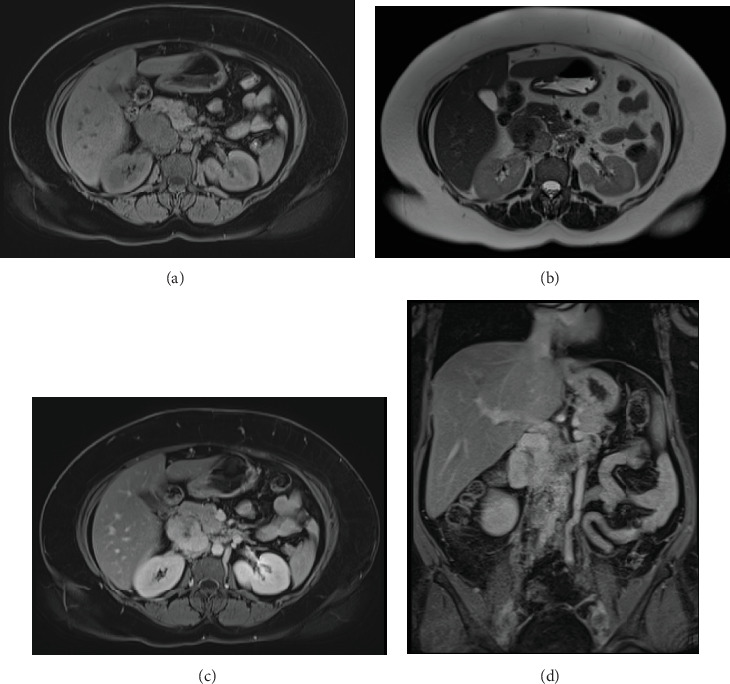
MRI abdomen. Nonenhanced (a) T1 and (b) T2 axial images show an irregular mass within IVC. The mass is best appreciated on postgadolinium-enhanced (c) axial and (d) coronal T1 images, where it appears as a heterogeneously enhancing retroperitoneal mass originating from the IVC with extraluminal extension.

Due to initial suspicions for a pancreatic neoplasm, a magnetic resonance cholangiopancreatography (MRCP) was performed. This demonstrated a 5 cm heterogeneously enhancing retroperitoneal mass within the middle segment of inferior vena cava (at the level of renal veins) with extracaval retroperitoneal infiltration, causing mass effect on duodenum and pancreatic head (Figure 2). Two small liver metastases were identified in Segment VI.

**Figure 3:**
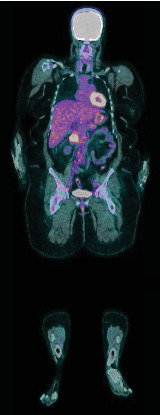
PET scan showing intense avidity in the known IVC neoplasm.

The diagnosis of LIVC was confirmed via endoscopic ultrasound–guided biopsy. PET scan demonstrated an intensely avid IVC neoplasm, suggestive of a high-grade neoplastic lesion (Figure 3). No new metastases were identified.

The consensus from the initial multidisciplinary team meeting (MDTM) was for neoadjuvant chemotherapy (ifosfamide and doxorubicin). Following the three cycles of chemotherapy, the patient had a restaging CT scan, which demonstrated disease progression. After rediscussion at MDTM, she proceeded to an IVC resection, right nephrectomy and liver excisional biopsy of Segment VI. Reconstruction of IVC was not required. Postoperatively, she had an uncomplicated recovery and was discharged on Day 10 of admission.

**Figure 4:**
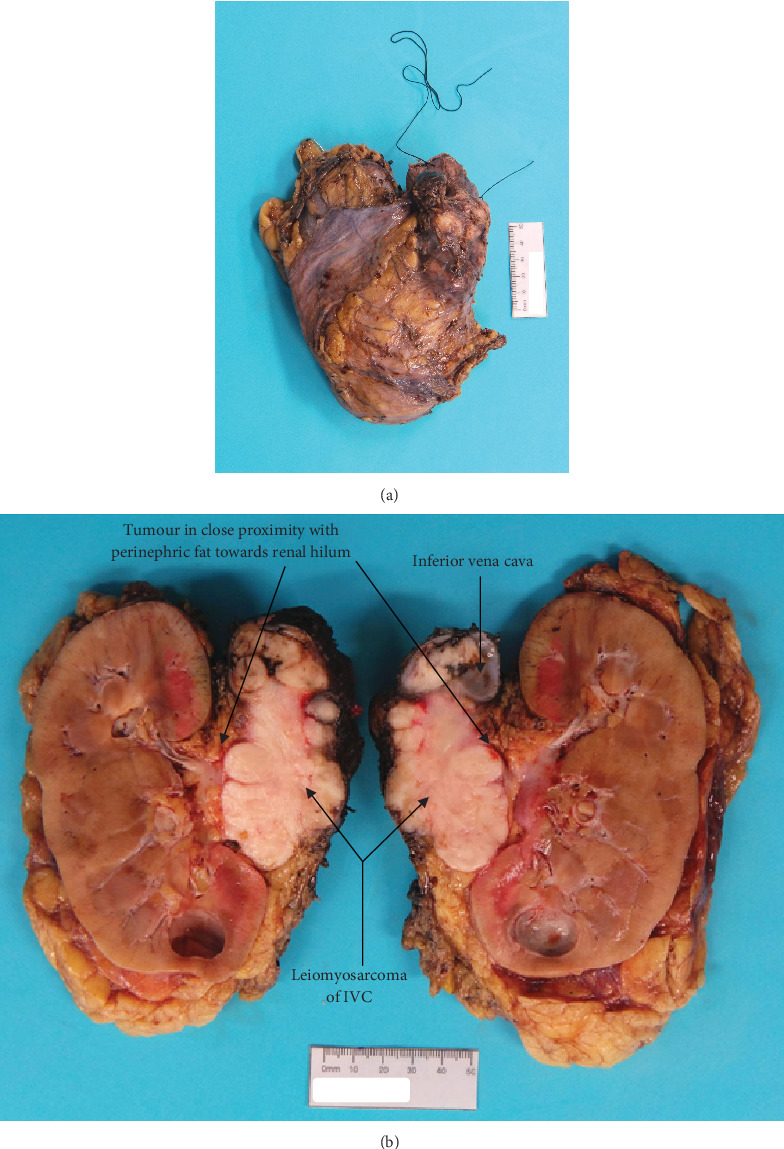
Gross macroscopic images of leiomyosarcoma of IVC. (a) The intact specimen consists of the IVC (long suture), right kidney and left renal vein (short suture). (b) The longitudinally bisected specimen shows a multilobulated circumscribed lesion abutting and arising from the IVC. The tumour is in close proximity with perinephric fat towards the renal hilum but does not appear to involve renal parenchyma.

Pathology reported a high-grade leiomyosarcoma of the IVC wall with extension into the lumen (Figure 4), measuring 80 mm in maximum dimension. The tumour infiltrated perirenal fat and renal vein, without renal parenchymal invasion. The liver excision confirmed metastatic leiomyosarcoma. These findings concluded an AJCC ypTNM stage of pT3N0M1, Stage IV.

Following surgical discharge, she was followed-up by medical oncology with surveillance CT scans every 3 months. There was no evidence of recurrence on the most recent imaging (6 months after surgery).

## 3. Discussion

LIVCs are rare malignant tumours arising from smooth muscle cells lining the blood vessel walls. They are an aggressive cancer with poor prognosis due to delays in presentation, complex surgical requirements, limited response to chemoradiotherapy and high recurrence rates [2, 6]. Wachtel et al. [7] and Mingoli et al. [8] published case series of 360 and 218 patients, respectively, and found that LIVC showed female predominance (3:1) and a median age of 55 years at time of diagnosis. Metastatic disease is common, being present in approximately 50% of patients upon diagnosis [3].

**Figure 5:**
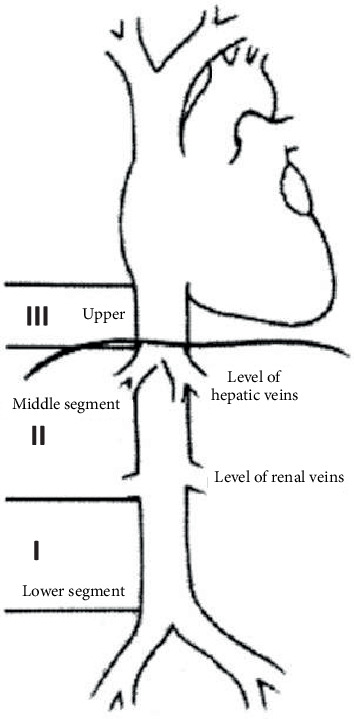
Classification of IVC leiomyosarcoma by segments, first described by Kulaylat et al. and image obtained from Teixeira et al [9].

LIVCs are classified based on location along the inferior vena cava—lower, middle and upper segments (Figure 5). The lower segment extends from the bifurcation of the IVC to below the renal veins; middle segment involves renal veins up to (but not including) hepatic veins; upper segment begins at hepatic veins and ends in the right atrium [6]. Wachtel et al. [7] found the majority of LIVC to be located in the middle segment (50.7%), followed by lower segment (22.5%) and upper segment (5.5%), and in 21.3% of cases, multiple segments were involved. LIVCs can also be categorised based on growth patterns: extraluminal (62%), intraluminal (5%) and mixed extra- and intraluminal (33%) [5].

### 3.1. Clinical Presentation

Clinical features of LIVC depend on the location and growth pattern of the tumour. Lower segment leiomyosarcomas may be asymptomatic or present with nonspecific symptoms, such as lower back pain and weight loss or lower limb oedema due to IVC obstruction [5, 7, 8]. Middle segment masses present with chronic abdominal pain or a palpable abdominal mass. Infrequently, they could develop nephrotic syndrome due to renal vein occlusion. Upper segment tumours are frequently complicated by Budd–Chiari syndrome (due to tumour obstruction of hepatic veins) or cardiac symptoms [5]. Mingoli et al. [8] found through their case series that the most common presenting complaints were abdominal pain (67%), palpable abdominal mass (42.7%), lower limb oedema (35.3%) and Budd–Chiari syndrome (16.5%).

### 3.2. Imaging Findings

Imaging plays a crucial role in diagnosis and management. Important radiological features to consider include tumour size, segment of IVC affected, extra/intraluminal involvement, relationship to surrounding structures and presence of metastasis [3, 5].

Ultrasound may demonstrate a heterogeneous hypoechoic retroperitoneal mass with intralesional vascularity on Doppler assessment [10], or abnormal IVC patency due to an obstructing intraluminal mass [5]. Ultrasound assessment is, however, frequently limited by body habitus and bowel gas [11].

CT is the modality of choice for staging, preoperative planning and surveillance. As with any oncological workup, a multiphasic protocol is recommended to characterise the leiomyosarcoma and evaluate surrounding vasculature. CT findings include a heterogeneously enhancing soft tissue mass arising from IVC [11]. The heterogeneity may be due to internal haemorrhage or necrosis, in which acute haemorrhages appear as increased attenuation on precontrast CT [5]. Intraluminal involvement may demonstrate an irregularly enhancing intraluminal mass obstructing and extending along the IVC course. Extraluminal LIVCs can be difficult to distinguish from other primary retroperitoneal masses on CT. Key features supporting LIVC are an imperceptible IVC at the point of maximal contact with the retroperitoneal mass, which has a sensitivity and specificity of 75% and 100%, respectively [11]. Radiologists should also assess the adequacy of venous collateral circulation as it would influence the surgical approach to the resection of the LIVC [5].

MRI is advantageous in evaluating sarcomas because of its superior tissue contrast, better definition of tumour boundaries and assessment of collateral circulation [3, 11]. On MRI, leiomyosarcomas appear hypointense or isointense relative to muscles on T1-weighted images and hyperintense relative to muscles on T2 [5]. On postgadolinium T1 images, LIVCs show heterogeneous enhancement. Contrast-enhanced MRIs demonstrate tumour morphology, extent of IVC involvement, collateral vessels, as well as differentiating tumour from non-neoplastic IVC thrombus [3]. On MRI, IVC luminal patency is demonstrated by signal void on spin echo sequences, which enhances less than the tumour. An advantage of MRI over CT is that LIVC metastases appear similarly with primary neoplasm [8].

PET scans are performed to grade tumour activity, look for distant metastases and aid prognostication [2]. The higher the avidity of the tumour, the higher the grading [12].

### 3.3. Diagnosis

It is essential to obtain a pretreatment biopsy to confirm the diagnosis and guide therapy. Radiology offers a minimally invasive option via CT-guided percutaneous biopsy of the extraluminal component of the neoplasm or transvenous approach for tumours with an intraluminal growth pattern. An alternative method would be endoscopic ultrasound–guided biopsy.

There are a number of differential diagnoses to consider when faced with an inferior vena cava tumour. As part of radiological evaluation, it is important to determine whether the mass originated from the retroperitoneum, a retroperitoneal organ or a primary LIVC. Solid organ tumours, which may involve the IVC, include renal cell carcinoma, hepatocellular carcinoma, adrenal cortical carcinoma and uterine leiomyosarcoma [5]. Renal cell carcinomas are the most common malignancy that extends into the IVC, often appearing as a heterogeneously enhancing renal mass invading through the renal vein and into IVC in 4%–10% of cases [5]. The radiologist should also consider primary retroperitoneal neoplasms, such as retroperitoneal leiomyosarcomas, leiomyomas, liposarcomas, neurogenic tumours and fibroblastic tumours [2, 5, 10].

### 3.4. Treatment

Following diagnosis of LIVC, a multidisciplinary team approach to guide therapy is highly recommended. Treatment options include neoadjuvant therapy, surgical resection and adjuvant therapy. Evidence for neoadjuvant chemoradiotherapy is limited, and current data has not shown any benefits towards prognosis or survival rates [5, 7, 8]. Interestingly, the LMS-04 study by the French Sarcoma group [13] has found that the addition of trabectedin to doxorubicin in first-line treatment of (uterine and soft tissue) leiomyosarcomas improved progression-free survival in patients with unresectable disease or metastases, when compared to doxorubicin-only therapy [3]. There is promising evidence for trabectedin to be used in LIVC refractory to ifosfamide and anthracyclines or unresectable cases [14].

Surgical approach is dependent on the segment of IVC affected, and nearby organs are often resected to achieve complete clearance. Following surgical resection of primary tumour, the IVC may be ligated, undergo primary repair or be reconstructed [2]. Upper segment LIVCs are generally inoperable, but if considered, involvement of cardiac surgeons is recommended due to the need for sternotomy access and cardiopulmonary bypass [3].

Postoperatively, adjuvant therapy can be considered to minimise risk of local recurrence; however, the survival benefits from adjuvant therapy are yet to be proven [7, 8]. There are no current guidelines recommending surveillance frequency; however, 3-monthly surveillance CTs in the first 2 years following initiation of treatment would be reasonable.

### 3.5. Prognosis

Despite advances in treatment, LIVC continues to have poor prognosis due to high rates of local and distant recurrences [1, 3]. Metastatic spread is usually haematogenous, commonly to the liver, lung, bone and lumbar spine. Following surgical resection, combined local and metastatic recurrence rate is 55%, with only 5% recurring locally without distant metastasis [1]. Some studies quote 6%–31.4% disease-free survival rate at 5 years with an overall survival rate at 5 years to be 49.4%–55% [7, 12]. Middle segment LIVCs have a good prognosis due to earlier presentations, which could be related to its mass effect on sensitive nearby organs [5, 7]. Poor prognostic factors include upper segment involvement, lower limb oedema and Budd–Chiari syndrome. The median overall survival time was 23 months; however, upper segment leiomyosarcomas had an average survival time of only 1 month [8].

## 4. Conclusion

The case encourages radiologists to consider a primary inferior vena cava neoplasm in the differential diagnoses for a retroperitoneal mass. Imaging plays a pivotal role in the assessment of leiomyosarcomas, preoperative planning and prognostication. It is important to consider the tumour characteristics, extent of IVC involvement and its relationship to surrounding structures and vasculature.

## Data Availability

Data sharing is not applicable to this article as no datasets were generated or analysed during the current study.

## Nomenclature

IVC: inferior vena cava
LIVC: leiomyosarcoma of inferior vena cava
CT: computed tomography
MRI: magnetic resonance imaging
PET: positron emission tomography
